# Use of an artificial intelligence-driven software device to assist endovascular repair of a ruptured abdominal aortic aneurysm

**DOI:** 10.1016/j.jvscit.2026.102288

**Published:** 2026-04-30

**Authors:** Theodorus G. van Schaik, Vinamr Rastogi, Patrick W.H.E. Vriens, Maarten K. Dinkelman, Marmix W. De Fijter, Jan M.M. Heyligers

**Affiliations:** Department of vascular surgery, Elisabeth Tweesteden Ziekenhuis, Tilburg, The Netherlands

**Keywords:** Endovascular aneurysm repair, Ruptured abdominal aortic aneurysms, Image fusion, Artificial intelligence software

## Abstract

Endovascular aneurysm repair for ruptured abdominal aortic aneurysms (RAAAs) is technically demanding due to time pressure and distorted vascular anatomy. Cydar Maps artificial intelligence-driven software integrates preoperative computed tomography angiography with live fluoroscopy to enhance procedural guidance. Its application in RAAAs has been limited because of perceived time delays. Yet, this technology could potentially shorten procedure time and reduce radiation exposure and contrast volume. The latter is particularly relevant, given the high incidence of acute kidney injury following RAAAs. This case demonstrates that Cydar Maps was feasible and safe during endovascular aneurysm repair for RAAAs in a hemodynamically stable patient.

Endovascular aneurysm repair (EVAR) is widely accepted as the preferred treatment for ruptured abdominal aortic aneurysms (RAAAs) in patients with suitable anatomy, due to improved survival compared with open repair.^1^^,^^2^ Despite progress in endovascular techniques, EVAR in the setting of rupture remains technically demanding and is associated with substantial morbidity and mortality.^3^ Acute kidney injury is common after rupture, largely because of hemodynamic instability and hypoperfusion.^4^^,^^5^

Image fusion (IF) technologies were introduced to improve accuracy during EVAR by integrating preoperative computed tomography angiography (CTA) with live fluoroscopy to generate a patient-specific three-dimensional vessel overlay of the aorta.^6^ Despite reducing procedure time, radiation exposure, and contrast use, the use of IF is limited in RAAAs.^7^^,^^8^ The main hesitation is the additional time needed to generate and align the vessel overlay, which may delay life-saving treatment. Yet the potential contrast reduction is highly relevant in patients with RAAAs who are at increased risk of contrast-induced nephropathy.

Our institution uses Cydar Maps (Cydar Medical), a cloud-based artificial intelligence (AI) software device, to assist endovascular treatment of patients, including the provision of intraoperative IF.^9^ A thin-slice CTA of the chest, abdomen, and pelvis is uploaded to the secured cloud and processed into a vessel overlay within approximately 30 minutes.^10^

Branch vessels are automatically identified and can be adjusted at any time if necessary. Typically, angiography is performed after guidewire insertion. The outline of the aorta and relevant orifices of side branches are aligned with the patient’s anatomy using a remote control. The overlay is aligned using vertebral landmarks; the software continuously monitors the display and automatically updates for patient position. By using the fluoroscopically viewed landmarks and not the table position as a frame of reference, the adjusted registration of the region of interest remains accurate and reliable throughout the procedure, even during procedural guidance in awake or agitated patients undergoing emergency EVAR under local anesthesia.

This case report describes a successful EVAR for an RAAA with assistance of Cydar Maps. Written consent for the current publication was obtained.

## Case report

A 69-year-old man with hypertension, intermittent claudication, and a history of coronary artery bypass presented in September 2025 after a collapse. He reported severe left lower abdominal pain, diaphoresis, and several episodes of transient loss of consciousness. He was hemodynamically stable, his blood pressure was 116/95 mmHg, his pulse was 90 bpm, and his Glasgow Coma Scale was 15.

Physical examination revealed a painful pulsatile abdominal mass. Laboratory values showed hemoglobin of 6.7 mmol/L and serum creatinine of 101 μmol/L.

**Fig 1:**
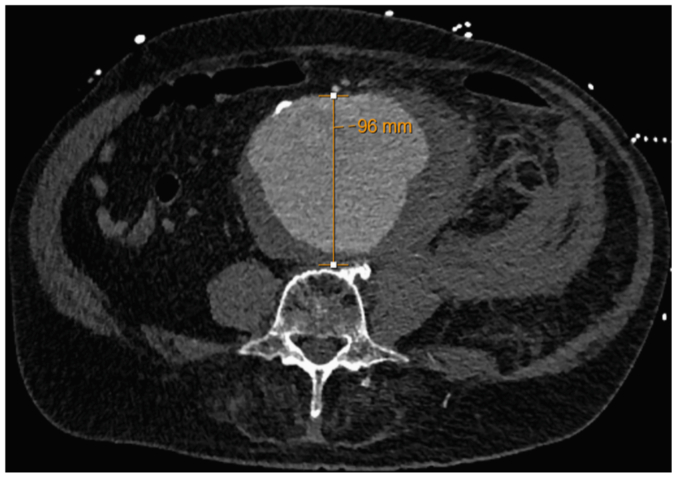
Computed tomography angiography (CTA) of the aneurysm rupture. Displayed is a maximal diameter of 96 mm. The retroperitoneal hematoma is visible in the left flank.

Ultrasonography demonstrated a large abdominal aneurysm suspicious for rupture without free intraperitoneal fluid. CTA confirmed a retroperitoneal contained rupture of a 96-mm diameter infrarenal abdominal aortic aneurysm with minimal thrombus and a flow lumen of 88 mm (Fig 1). Both internal iliac arteries were occluded. The aneurysm characteristics are summarized in Table I.

**Table I:** Aneurysm characteristics

Aneurysm severity grading score	
Aneurysm neck length, mm	31
Aneurysm neck angle	22°
Aneurysm neck diameter, mm	24
Aneurysm neck calcification and/or thrombus	8%
Maximal aneurysm diameter, mm	96
Patent mesenteric vessels	3 (celiac trunk, SMA, and IMA)
Left CIA diameter, mm	12
Right CIA diameter, mm	11
Left CFA diameter, mm	10
Right CFA diameter, mm	10
Iliac calcification	Minimal
Intraluminal thrombus	Minimal
Maximal flow lumen, mm	88

*CFA*, common femoral artery; *CIA*, common iliac artery; *IMA*, inferior mesenteric arter; *SMA*, superior mesenteric artery.

**Fig 2:**
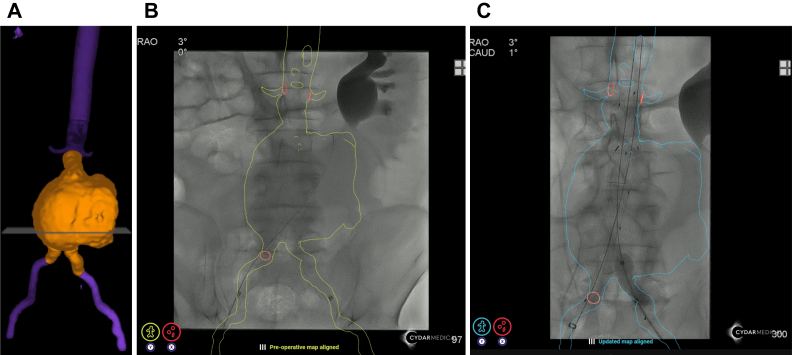
**(A)** Three-dimensional aneurysm anatomy. Displayed is the reconstructed three-dimensional representation by Cydar EV Maps; this tool also enables automated volume measurement of the aneurysm. The aneurysm sack is displayed in *orange*, and the suprarenal aorta and iliac vessels are displayed in *purple*. **(B)** Cydar EV Maps vessel overlay. Displayed is the automated rigid vessel overlay during surgery prior to angiography. Computer vision continuously watches the X-ray images, and after identifying two or more vertebrae, an automated overlay appears on the screen. An indication of the outer lines of the aneurysm is in *yellow*, and the ostia of the aortic branch vessels is in *red*. **(C)** Cydar EV Maps vessel overlay. The physician-adjusted vessel overlay after angiography is shown. It is defined while having the C-arm in the position with a perpendicular view on the orifice of the lowest renal artery. An indication of the outer lines of the aneurysm is displayed in *blue*, and the ostia of the aortic branch vessels is shown in *red*. Note that the level of the renal orifices is slightly different from the automated overlay due to the adjustments. This picture is after successful exclusion of the aneurysm, ballooning the endovascular aneurysm repair (EVAR) device.

The patient was admitted to the intensive care unit (ICU) for blood pressure monitoring with permissive hypotension according to Dutch guidelines, given his hemodynamic stability and concurrent use of the preferred hybrid operating theater for an elective endovascular procedure. This provided the opportunity to upload the CTA to the Cydar Cloud and generate the three-dimensional overlay for perioperative use (Fig 2). The preparation process takes about 25 to 30 minutes.

### Procedure

Without hemodynamic problems, the patient arrived after 2 hours and 25 minutes to the hybrid operation theater. EVAR started approximately 2.5 hours after confirmation of the diagnosis and was performed under local anesthesia. Both common femoral arteries were accessed percutaneously under ultrasound guidance, with percutaneous wire insertion, after which two Perclose ProGlide system closure devices (Abbott Vascular) were placed in each groin and successfully used to close the vessels at the end of the procedure. The patient remained awake, cooperative, and hemodynamically stable throughout the procedure, and the use of an available occlusion balloon (resuscitative endovascular balloon occlusion of the aorta technique) was not required.^11^ Operative parameters are listed in Table II.

**Table II:** Operative results

Anesthesia	Local (10 mL 2% lidocaine per groin)
Ipsilateral sheath (right), Fr	18
Contralateral sheath (left), Fr	12
Closure device (ipsilateral)	double Perclose ProGlide (Abbott Vascular)
Closure device (contralateral)	double Perclose ProGlide (Abbott Vascular)
Main device	Gore Conformable Excluder 261412
Ipsilateral limb	Gore 141000
Contralateral limb	Gore 141400
Operating time	1 hour 40 min
Contrast volume	91 mL jodixanol 320 mgi/mL
Radiation dose, mGy	609.65
Radiation time, minutes	36
Total dose area product, Gycm^2^	126
Blood loss, mL	200

After the initial angiogram, the overlay was aligned to the renal artery orifices using remote control and remained unchanged throughout the procedure, as these were the sole regions of interest given pre-existing occlusion of both internal iliac arteries. Consequently, the rest of the anatomy was not adjusted, resulting in wires and catheters being virtually projected ‘outside’ the vasculature (Fig 2). A Gore Excluder Conformable (W.L. Gore and Associates) main body was deployed just below the lowest (left) renal artery. Contralateral limb cannulation was challenging due to the large flow lumen but was successfully achieved. Because both internal iliac arteries were already occluded, limb extensions were placed without additional angiography in a cross-limbed configuration. Completion angiography confirmed accurate positioning without endoleaks; the procedure time was 1 hour and 40 minutes. The contrast volume used was 91 mL jodixanol 320 mgi/mL, the radiation time was 36 minutes, and the radiation dose was 609.65 mGy.

### Postoperative recovery

The patient returned to the ICU, where 12 hours later, he developed recurrent left flank pain and a hemoglobin drop from 6.5 to 4.6 mmol/L. CTA showed a type II endoleak originating from the inferior mesenteric and sacral arteries, without clinical sequelae.

As he remained stable, conservative management was initiated: optimization of coagulation, analgesia, and transfusion of red cells and platelets. Hemoglobin stabilized over 48 hours, and his pain resolved. Serum creatinine rose transiently to 159 μmol/L but normalized with supportive therapy. He was transferred to the surgical ward on postoperative day 2 and discharged home on day 7 with serum creatinine at 105 μmol/L.

One-month follow-up CTA demonstrated aneurysm sac regression from 96 to 92 mm without complications or persistent endoleaks.

## Discussion

This case illustrates the safe use of an AI-driven software technology to provide intraoperative IF during EVAR for an RAAA in a hemodynamically stable patient. The use did not delay surgery, as the 25- to 30-minute processing period occurred while the patient was monitored in the ICU. Once surgery started, no further steps were required for precise procedural guidance, as the registration is automatic, unlike mechanically registered IF systems. Thus, AI-based IF can potentially narrow the gap between elective and emergency workflows, provided the patient is sufficiently stable to allow overlay generation. Only a few prior reports describe successful use of other IF systems in rupture scenarios.^12^^,^^13^

In elective EVAR, IF has consistently been shown to reduce radiation, fluoroscopy time, contrast use, and, in some cases, overall procedure time, particularly in complex repairs.^14^^,^^15^ In our case, contrast usage was minimal, and fluoroscopy and operative time were as expected, with any prolongation from contralateral limb cannulation, rather than the use of this IF technology in an emergent situation.

Reduced contrast administration is especially relevant because renal impairment is more common after emergency than elective repair.^3^^,^^4^ A recent report described generating an overlay using only a noncontrast computed tomography in a patient with tamponaded rupture, thereby further decreasing contrast exposure.^13^

A potential advantage of this AI software is its frame of reference. Unlike table-based systems, this technology uses a computer vision machine learning algorithm with the patient’s vertebrae as the frame of reference to register the anatomy on fluoroscopy. It calculates deformation from patient movement and introduction of stiff wires and devices, providing real-time vessel overlay correction without angiographic reregistrations. This potentially reduces contrast use and procedure time in emergent EVAR, particularly in awake or agitated patients. Stable and reliable anatomical overlays may also aid in accurate renal artery localization even when hypotension or hematoma obscure intraluminal flow on fluoroscopy. This could support precise balloon occlusion—in the case of hemodynamic instability—or stent graft deployment.

A potential limitation is overlay inaccuracy if the rupture causes substantial vascular deformation or if the preoperative CTA is insufficient (low contrast or inadequate timing), complicating aortic side branch detection. Therefore, high-quality, thin-slice CTA is preferred for successful use of Cydar Maps.

The type II endoleak occurrence is unrelated to the use of this AI-driven IF technology. This system does not aim to reduce or alter treatment for type II endoleaks. However, by improving accuracy at the aortic and iliac bifurcations, it theoretically may enhance sealing and reduce endoleaks related to inadequately covered common iliac arteries.

The successful workflow in this case highlights the importance of coordination between departments. Immediate transfer of CTA data after diagnosis allows generation of the AI-assisted vessel overlay during preparation of the hybrid theater, avoiding delay. Because our patient was hemodynamically stable, this case cannot be extrapolated to all patients with RAAAs. The use of this AI-driven IF solution in RAAAs should be limited to hemodynamically stable patients only. Because the setup of this technology is dependent on a cloud-based process, technical problems could delay intervention. Therefore, any procedural delay from IF setup remains unacceptable in unstable patients.

Future research must clarify which patients with RAAAs benefit most from IF. The ongoing ARIA trial may help determine its effect on procedural efficiency, radiation dose, and renal outcomes.^16^ Larger multicenter studies should stratify results by rupture morphology and hemodynamic status to define the role of IF in emergent EVAR.

## Conclusions

This case demonstrates that the Cydar Maps AI software technology can be safely integrated into RAAA treatment in a hemodynamically stable patient. Automatic display of a three-dimensional vessel overlay facilitated fast and accurate deployment without delaying intervention.

## Funding

None.

## Disclosures

None.
